# Development, validation and evaluation of a rapid PCR-nucleic acid lateral flow immuno-assay for the detection of *Plasmodium* and the differentiation between *Plasmodium falciparum* and *Plasmodium vivax*

**DOI:** 10.1186/1475-2875-11-279

**Published:** 2012-08-17

**Authors:** Petra F Mens, AntoinePHA Moers, Laura M de Bes, Jonathan Flint, Jathee R s Sak, Lily Keereecharoen, Chantal van Overmeir, Jaco J Verweij, Rachel L Hallett, Benchawan Wihokhoen, Stephane Proux, Henk DFH Schallig, Aart van Amerongen

**Affiliations:** 1 Koninklijk Instituut voor de Tropen / Royal Tropical Institute, Biomedical Research, Meibergdreef 39, 1105 AZ, Amsterdam, The Netherlands; 2 Wageningen UR Food & Biobased Research, Biomolecular Sensing & Diagnostics, Wageningen, The Netherlands; 3 Forsite Diagnostics Ltd, Sand Hutton, York, YO41 1LZ, UK; 4 Shoklo Malaria Research Unit, Mae Sot, Thailand; 5 Mahidol-Oxford Tropical Medicine Research Unit, Mahidol University, Bangkok, Thailand; 6 Instituut voor Tropische Geneeskunde, Department of Biomedical Sciences, Nationalestraat 155, 2000, Antwerp, Belgium; 7 Department of Parasitology, Leiden University Medical Center, PO Box 9600, 2300 RC, Leiden, The Netherlands; 8 London School of Hygiene and Tropical Medicine, Faculty of Infectious and Tropical Diseases, Keppel Street, London, WC1E 7HT, UK

## Abstract

**Background:**

Molecular tools are very sensitive and specific and could be an alternative for the diagnosis of malaria. The complexity and need for expensive equipment may hamper implementation and, therefore, simplifications to current protocols are warranted.

**Methods:**

A PCR detecting the different *Plasmodium* species and differentiating between *Plasmodium falciparum* and *Plasmodium vivax* was developed and combined with a nucleic acid lateral flow immuno-assay (PCR-NALFIA) for amplicon detection. The assay was thoroughly evaluated for the analytical sensitivity and specificity in the laboratory, the robustness and reproducibility in a ring trial and accuracy and predictive value in a field trial.

**Results:**

The analytical sensitivity and specificity were 0.978 (95% CI: 0.932–0.994) and 0.980 (95% CI: 0.924-0.997), respectively, and were slightly less sensitive for the detection of *P. vivax* than for *P. falciparum.* The reproducibility tested in three laboratories was very good (k = 0.83). This evaluation showed that the PCR machine used could influence the results. Accuracy was evaluated in Thailand and compared to expert microscopy and rapid diagnostic tests (RDTs). The overall and *P. falciparum*-specific sensitivity and specificity was good ranging from 0.86-1 and 0.95-0.98 respectively, compared to microscopy. *Plasmodium vivax* detection was better than the sensitivity of RDT, but slightly less than microscopy performed in this study.

**Conclusion:**

PCR-NALFIA is a sensitive, specific and robust assay able to identify *Plasmodium* species with good accuracy. Extensive testing including a ring trial can identify possible bottlenecks before implementation and is therefore essential to perform in additon to other evaluations.

## Background

The correct and timely diagnosis of life-threatening diseases such as malaria is essential for the initiation of accurate and prompt treatment [1]. However, correct diagnosis of infectious diseases is often difficult in resource-poor settings, resulting in clinical decisions based on presumptive diagnosis [2]. This can lead to unnecessary over-treatment of one disease and under-recognition of another [3]. The diagnosis of malaria is often complicated by the fact that routine microscopy has a relatively low detection limit, is time-consuming and requires experienced microscopists to obtain reliable results [4]. Rapid diagnostic tests (RDTs) could be a good alternative but the detection limit is currently even lower than microscopy, especially when the infecting agent is non-falciparum. RDTs specifically detecting *Plasmodium vivax* for instance can have a very low sensitivity [5,6].

Molecular diagnostic tools, such as PCR, are highly sensitive and specific but involve sample processing. The read-out of the assay requires either laborious gel-electrophoresis, which uses toxic products and UV-light or expensive equipment to measure the assay in real time [7]. Simplifying this methodology is thus warranted and has been partly achieved by using, for example, isothermal amplification technologies such as LAMP [8] or simple and cheap read-out systems, such as nucleic acid lateral flow immuno-assay (NALFIA) [9,10], which can circumvent the use of electrophoresis or the purchase of expensive real-time PCR machines. NALFIA is analogous to an RDT but instead of detection of antigens it will detect amplified nucleic acids. Like an RDT the sample pipetting is fast, the results can be kept for further reference after the test is performed and the waiting time before the result can be visually read is only 10 minutes without the need of UV light and ethidium bromide.

Essential to the development of a new diagnostic test is determining the accuracy in diagnosing the right condition [11]. Therefore, high sensitivity (the probability of a positive test result for a patient with the disease) and specificity (the probability of a negative test for a patient that does not have the disease) is required [12]. All new diagnostic tests need to be assessed for these characteristics. This entails a comparison with the gold or reference standard that should be infallible. This is difficult in the case of malaria because the obvious reference standards, microscopy and RDT, which are routinely used in the field, are not as sensitive as molecular tools, leading to an underestimation of the specificity of the molecular assay.

Nevertheless, in order to estimate the accuracy of a diagnostic test it has to undergo different testing phases [13,14]. During the first phase a small study is performed on typical patients with and without the disease of interest, and the analytical sensitivity and specificity is assessed. If it fails this phase than the test is not worth further exploring without significant modifications. During the second evaluation phase in which the diagnostic sensitivity and specificity are assessed, a large number of patients are tested. The patient sample should be representative of the target population. Although these results might give a good reflection of the sensitivity and specificity, it is also essential to study the reproducibility and ruggedness of a test when other operators perform the assay. Although with the ideal test the performance is not operator-dependent, many tests show variation when performed in different laboratories [14]. Having the test performed by different operators and/or different laboratories will reveal whether the test is reproducible or not [15], and whether a large rollout would be a feasible aim. This can be studied in a so-called ring trial.

This study describes the development of a species-specific PCR combined with NALFIA for the detection of pan*-Plasmodium* species and the discrimination between *Plasmodium falciparum* and *P. vivax.* In addition the test has been evaluated for the accuracy in an exploratory study in the laboratory and thereafter validated in Mea-Sot Thailand for accuracy in the final target population. Reproducibility was assessed by evaluating the assay in a ring trial in three different laboratories that did not participate in the development of the assay.

## Methods

### DNA isolation and PCR

DNA was extracted, with Qiagen DNA mini kit (Qiagen, Germany) according to the manufacturer’s instructions, from all the whole blood samples used in this study. For each sample 200 μl of whole blood was used and the samples were eluted in 200 μl TE buffer. The PCR reaction consisted of primers (Table 1), targeting the 18S gene of *Plasmodium* and the human housekeeping gene glyceraldehyde 3-phosphate dehydrogenase (GAPDH), which was used as an internal control. For the discrimination of two *Plasmodium* species, general pan-*Plasmodium* and specific *P. falciparum* and *P. vivax* forward primers were designed. As a reverse primer, a generic primer developed on a conserved region present in all three targets, was used. The PCR reaction with a final reaction volume of 25 μl comprised of 12,5 μl 1X Qiagen Multiplex PCR Master mix (Qiagen, Germany), 200 μM dUTP, 2% DMSO, 1U UNG (Fermentas), primers in the concentration as presented in Table 1, and 2 μl template DNA. The cycling conditions consisted of an nitial step of 10 min at 50°C followed by one step of 15 min at 95°C, 35 cycles of 45 sec at 94°C, 45 sec at 54°C and 60 sec at 72°C and a final step of 10 min at 72°C. For each experiment a blood sample negative for *Plasmodium*, a sample containing *P. falciparum,* a sample containing *P. vivax* and a non-template control of only water were used as control samples.

**Table 1:** Overview of the primer sequences and used concentrations in the PCR-NALFIA reaction

** Target**	**Primer type**	**Sequence**	**Product size (bp)**	**Concentration in PCR mix (nM)**
GAPDH	Forward	Biotin-5’ TGCACCACCAACTGCTTAGC -3’	90	75
GAPDH	Reverse	Texas Red-5’ GGCATGGACTGTGGTCATGAG -3’		75
Pan-*Plasmodium*	Forward	Digoxigenin-5’ TCAGATACCGTCGTAATCTTA -3’	180	50
Pan*-Plasmodium*	Reverse *	Biotin-5’- AACTTTCTCGCTTGCGCG -3’		900
*P. falciparum*	Forward	FAM-5’ GTCATCTTTCGAGGTGACTT -3’	100	200
*P. vivax*	Forward	DNP-5’ TTTCTCTTCGGAGTTTATTC -3’	100	650

* This primer was used as a reverse primer for the *P. falciparum* and *P. vivax* reaction as well.

**Table 2:** Overview of the origin and number of samples used in the laboratory (n = 241) and ring trial (n = 94) evaluations

**Origin of the samples**	** Sample type**	**Laboratory evaluation (n)**	**Ring trial (n)**
Wad Medani Teaching Hospital, Sudan	*P. falciparum*	72	6
Malaria Reference Laboratory, London School of Hygiene and Tropical Medicine, UK, returning travellers	*P. falciparum*	7	5
	*P. vivax*	26	9
	*P. ovale*	7	4
	*P. malariae*	18	4
	*P. falciparum/ P. vivax*	2	1
	*P. falciparum/ P. ovale*	5	
	Negative		18
Academic Medical Centre, The Netherlands, returning travellers	Negative	9	
Dutch Blood Bank	Negative	95	45
Culture of *P. falciparum*			2

### Nucleic acid lateral flow immuno-assay

A nitrocellulose membrane of 120 mm (Millipore, Amsterdam, The Netherlands) was used for the preparation of the NALFIA. On the nitrocellulose, 0.8 mg/ml anti-Texas Red rabbit IgG fraction (Invitrogen, Paisley, UK), 0.2 mg/ml anti-Digoxigenin polyclonal antibody (Roche Diagnostics, Mannheim, Germany), 0.4 mg/ml anti-dinitrophenyl- rabbit IgG fraction, (Invitrogen, Paisley, UK), and 0.1 mg/ml anti-FITC purified polyclonal antibody rabbit polyclonal IgG, (AbD Serotec, Oxford, UK) were sprayed, all at 0.8 μl/cm and within 4.5 mm distance of each other. A Surewick G041 glass-fibre sample pad (Millipore, Billerica, USA) was used to spray 2.5 μl/cm streptavidin-labelled carbon suspension (three parts streptavidin-labelled carbon and two parts sodium tetraborate buffer solution containing 6.25% sucrose and 6.25% BSA) and attached to the NALFIA stick. The strips were air dried at 37°C, packed in plastic housing and sealed in airtight bags containing silica until further use.

**Figure 1:**
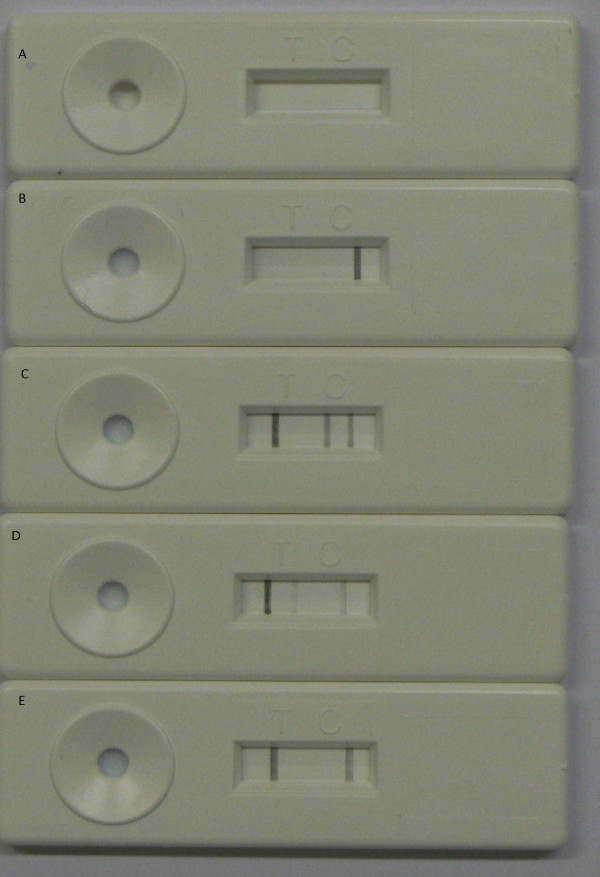
**Examples of the different test outcomes of the PCR-NALFIA.a**) No control and no test line are visible. This is a test failure but can also be seen when only water is amplified (negative control) **b**) A positive control line and a negative test line are visible indicating that the test is valid but that no parasite DNA is detected **c**) Next to the control a *Plasmodium* line and a specific line for *P. falciparum* is present indicating a *P. falciparum* infection **d**) Next to the control and *Plasmodium* line the specific line for *P. vivax* is visible indicating a *P. vivax* infection. **e**) Both a positive control and a positive test line for *Plasmodium* are visible indicating a valid test positive for *Plasmodium* but negative for *P. falciparum* and *P. vivax*.

After amplification 5 μl PCR product and 70 μl running buffer (0,1 M borate buffer pH 8.8, 1% BSA and 1% (v/v) sodium azide) were added to the sample pad of the NALFIA and after 10 min the results were read. The NALFIA was considered valid if a black line at the GAPDH position was visible. If in addition to the GAPDH line, the pan line was present then the sample contained *Plasmodium* DNA. If in addition the *P. falciparum* line was present then a *P. falciparum* infection was confirmed. The same held for the *P. vivax* line in combination with the pan-*Plasmodium* line. If all four lines were present than it was considered to be a mixed infection. If no line appeared on the GAPDH position, regardless of other lines that might have appeared, the NALFIA test was considered to be a test failure. An example of the different possibilities of the NALFIA outcomes is shown in Figure 1.

### Laboratory evaluation PCR-NALFIA

The analytical sensitivity of the PCR-NALFIA test was assessed by using a 5% *P. falciparum* NF54 ring stage culture (in 5% haematocrit). The cultured parasites were 10-fold diluted with *Plasmodium*-negative donor blood to obtain malaria parasites dilutions from 0.55% to 5x10^-8^% in 45% haematocrit. The specificity of the test was determined by analysing malaria positive and negative samples (n = 241) from: i) travellers returning from malaria-endemic areas, diagnosed by an experienced technician by microscopy and/or *Plasmodium-*specific nested PCR according to[ Snounou *et al.* 16]; ii) from Sudanese patients with a confirmed *P. falciparum* infection; and, iii) samples provided by the Dutch blood bank which excludes donations of patients who travelled in the past nine months to malaria-endemic areas and can therefore be considered negative for malaria (Table 2).

### Inter-laboratory evaluation PCR-NALFIA

In order to assess the inter-laboratory variability of the assay, a diagnostic ring trial was performed. All necessary reagents and 94 processed samples were sent to three laboratories that were not directly involved in the development of the current PCR and NALFIA technology. These materials were accompanied by a protocol on how to perform the assay but no further training was given. The laboratories used their own PCR equipment and the assay was performed by personnel who had previous experience performing PCR. In addition to the patient samples presented in Table 2 two DNA samples of pure *P. falciparum* culture of 10 and 1 p/μl were provided. The protocol indicated that two non-template controls should be added by the laboratory performing the assay. The technicians performing the assay were blinded as to the contents of the samples and the performance of the other laboratories in the trial.

### Field evaluation of PCR-NALFIA, study site and patient description

A prospective study was conducted in the health clinics of Wang Pa and Mae Khon Ken, Mae Sot district, Tak Province, Thailand, which is located on the Thai-Myanmar border. In this hill-forested region, with low year-round transmission and two seasonal peaks from May-September during the rainy season and December-January, the predominant species causing malaria are *P. vivax* and during the rainy season *P. falciparum*[17]. Patients were recruited in the malaria clinics of Wang Pa and Mae Khon Ken located around Mae Sot. Samples were transported to the Shoklo Malaria Research Unit (SMRU) in Mae Sot for further processing. Ethical approval for the evaluation in Thailand was obtained from the local ethical review board, Mahidol Oxford Research Unit and the Oxford Tropical Research Committee. Patients visiting the outpatient clinics with the clinical suspicion of uncomplicated malaria, over three years old, and willing to participate in the study, were enrolled after obtaining their informed consent or in the case of minors that of their parents or guardians. Additional inclusion criteria were axillary temperature ≥ 37.5°C or a history of fever in the previous 24 hours.

From each participant, finger prick blood was collected in EDTA tubes (Stastedt, Numbrecht, Germany) for preparation of thin and thick Giemsa-stained microscopy slides, to perform a HRP-2 and PAN pLDH-based RDT and 200 μl of blood was used for DNA isolation. Microscopy was performed according to international and GCP guidelines by local expert microscopists [18]. Parasites were counted against 500 leukocytes at 1,000x magnification. If only one parasite was found after counting of 500 WBC then counting continued until a second parasite was observed with a limit of counting 4,000 WBC. All slides were read by two microscopists and discordant results were read by a third reader. A leukocyte count of 8000/μL was assumed to calculate the parasite density per μl and the final result was the geometric mean of the readings. Peripheral blood was applied to the SD Bio Line Malaria Antigen P.f/Pan POCT RDT (Standard Diagnostics Inc) according to the procedures described by the manufacturer. The PCR-NALFIA was performed as described above. All test operators were blinded to each other’s test results. On the discordant results and a selection of the microscopy negative samples (n = 71) an additional diagnostic nested PCR was performed as described by Snounou *et al.*[16].

### Data analysis

Data from the laboratory evaluation and the ring trial were directly entered into Excel. Data from the field evaluation were collected on separate case record forms and subsequently entered in Excel. Calculations of sensitivity, specificity and agreement between microscopy, RDT and PCR-NALFIA were done using Epi Info version 6.04 (Center for Disease Control and Prevention, Atlanta, GA, USA). For the ring trial analysis a combined kappa was calculated by using the formula for more than two raters and more than two outcomes as described by [Fleiss *et al.* 19]. Each of the samples was rated into one of the six subtypes (*P. falciparum, P. vivax*, negative, *P. falciparum* and *P. vivax* mixed infection, non-*P. falciparum* and non-*P. vivax* infection or water controls) by the three laboratories. Kappa (κ) values express the agreement beyond chance and were calculated with a 95% confidence interval. Kappa was calculated for each of the subtypes separately against an amalgam of the remaining categories. For example, the *P. falciparum* kappa is the two-rating kappa where *P. falciparum* is positive and all remaining outcomes are negative. The combined kappa is the appropriately weighted average of the individual kappas. There is considerably more agreement about the rating of samples as being *P. falciparum* compared to *P. falciparum/P. vivax*. A ĸ-value [20] of 0.21-0.60 is a moderate, a ĸ-value of 0.61-0.80 a good and a ĸ-value >0.80 is an almost perfect agreement beyond chance. Kappas were calculated by STATA SE11.2

## Results

### Analytical performance of the PCR-NALFIA

The analytical sensitivity could only be assessed for *P. falciparum* as there was no reference sample with *P. vivax* available with a certified number of parasites. The analytical sensitivity was 1 p/μl for both the Pan-band and the *P. falciparum* band with the *P. falciparum* sample. The samples were also once run on agarose gel to confirm the expected amplicon size of both GAPDH and the *Plasmodium* fragments.

The phase 1 evaluation of 241 samples resulted in three test failures (two in negative controls and one *P. falciparum*-positive sample), meaning that no GAPDH line could be observed. Of the negative samples, five were found positive by PCR-NALFIA for *P. falciparum,* of which two were also positive in the pan-*Plasmodium* line. None of the negative samples reacted with the *P. vivax* line. All *P. falciparum* samples reacted with both the pan-*Plasmodium* and *P. falciparum* line, including the sample that had no GAPDH line. None of the samples reacted with the *P. vivax* line. All *P. vivax* samples were positive for pan-*Plasmodium* but three failed to react with the specific *P. vivax* line. None reacted with the *P. falciparum* line. Of the 18 *Plasmodium malariae* samples, three were negative and one indicated a *P. vivax* infection. All *Plasmodium ovale* samples, including the *P. falciparum/P.ovale* mixed infections were identified correctly. The *P. falciparum/P.vivax* mixed infection samples were all positive for pan-*Plasmodium* and *P. falciparum* but one failed to detect the additional *P. vivax* line.

To determine sensitivity and specificity, the test failures were excluded and microscopy results were used as the reference. The overall sensitivity and specificity for the detection of any species through the pan-*Plasmodium* line in this specific data set was 0.978 (95% CI: 0.932–0.994) and 0.980 (95% CI: 0.924-0.997) respectively. For the detection of *P. falciparum* through the specific *P. falciparum* line, the sensitivity and specificity in this set was 1 (95% CI: 0.946-1) and 0.967 (95% CI: 0.921–0.988). The sensitivity of the *P. vivax* line was lower in this evaluation (0.857 (95% CI: 0.664-0.953)) but the specificity was good (0.995 (95% CI: 0.969-0.999)).

### Intra-assay variability between three independent laboratories

Of the 96 samples that were tested by the three different laboratories, 84 were identified correctly by all three laboratories. Table 3 gives an overview of the score of correct answers per individual laboratory. One sample containing *P. falciparum* was scored as a test failure in all three laboratories. In addition, laboratory 1 identified two negative samples as a *P. falciparum* infection, one *P. vivax* sample as negative and another *P. vivax* sample as a mixed infection with *P. falciparum*. Laboratory 2 had only two additional misdiagnoses. One negative sample was interpreted as a non-*P. falciparum*/non-*P. vivax* sample and one *P. vivax* sample was interpreted as a mixed infection with *P. falciparum,* which was the same sample that was misdiagnosed with the same interpretation as laboratory 1. Laboratory 3 scored the least of the three laboratories. Three samples that were positive for either *P. falciparum*, *P. vivax* or *P. ovale* were scored negative. One negative sample was interpreted as positive for *P. falciparum*, a *P. vivax* and a *P. falciparum* sample were both identified as non-*P. falciparum*/non-*P. vivax* and a *P. falciparum/P. vivax* co-infection was interpreted as a *P. falciparum* mono-infection. Despite these discrepancies, the overall agreement between laboratories and the reference panel was good to almost perfect. The kappa amongst the different laboratories can be found in Table 4. The agreement between the different samples was also good and can be seen for the different species in Table 3. The overall kappa was 0.839. Only the *P. falciparum*/*P. vivax* mixed infection agreement is very low but this is caused by the fact that only one sample of this type was analysed.

**Table 3:** Results of the ring trial per sample type and laboratory

**Sample type**	**Laboratory 1**	**Laboratory 2**	**Laboratory 3**	**Kappa**
*P. falciparum*	12/13*	12/13*	10/13*	0.873
*P. vivax*	7/9	8/9	7/9	0.727
*P. ovale*	4/4	4/4	3/4	0.836^^^
*P. malariae*	4/4	4/4	4/4	
*P. falciparum/P. vivax*	1/1	1/1	0/1^$^	0.497
Negative^#^	61/63	62/63	62/63	0.858

^>^ Including two *P. falciparum* culture samples.

*One sample failed in all three laboratories.

^#^ The two water controls the laboratories added themselves are not presented in the table.

^$^ This sample was identified as a *P. falciparum* mono-infection.

^^^ The results for *P. ovale* and *P. malariae* samples were scored as positive, non-falciparum, non-vivax and are therefore combined in the analysis.

**Table 4:** **Levels of agreement between different laboratories in the ring trial for the detection and differentiation of*Plasmodium*species with PCR-NALFIA**

	K values*		
	**Laboratory 1**	**Laboratory 2**	**Laboratory 3**
**Reference**	0.90	0.94	0.84
**Laboratory 1**	-	0.92	0.80
**Laboratory 2**	-	-	0.76
**Laboratory 3**	-	-	-

*The k values represent the agreement beyond chance between each laboratory and the reference and between two laboratories.

### Field evaluation of the PCR-NALFIA in a malaria-endemic setting

The evaluation of the PCR-NALFIA took place between December 2011 and January 2012. In total, 381 patients were included of which 85 patients were seen in Mae Khon Ken and 296 in Wang Pa. The mean age of the participants was 20.6 years (range four to 63 years), 33% was female and 67% was male, which is representative for the region. In total, 274 of the patients recruited into the study were found to be negative with microscopy, 72 were found to be harbouring *P. vivax,* (mean parasitaemia 730 p/μl), 32 *P. falciparum* (mean parasitaemia 5185 p/μl) and three a mixed infection. A complete overview of the results can be found in Table 5. When RDT is compared to microscopy, RDT had four test failures and detected a substantially lower number of *P. vivax* cases than microscopy (14 of 72 microscopy confirmed *P. vivax* were missed). The PCR-NALFIA detected more positive samples than were found negative with microscopy. Three were identified as *P. falciparum*, six were identified as *P. vivax* and five others as a mixed infection. The 14 samples that were negative for *P. vivax* with PCR-NALFIA were the same that were negative with RDT. Seven of these samples had a parasitaemia below two parasites/ μl, four samples between four parasites/μl and 10 p/ul and three between 20 p/μl and 32 p/μl. The calculated sensitivity and specificity of the different detection lines of the PCR-NALFIA compared to the different assays can be found in Table 6. When only the microscopy positive samples are compared to the RDT and PCR-NALFIA results and stratified to parasitaemia, it is found that PCR NALFIA is able to reliably detect samples with a parasitaemia of over 50 p/μl whereas RDT is already having problems with identifying some of the samples below 500 p/μl, as can be seen in Table 7. When the discordant results were analysed with a nested PCR, the three PCR-NALFIA positive *P. falciparum* samples were confirmed (including the RDT positive sample) to be positive for *P. falciparum*; three of the *P. vivax* samples were confirmed to contain *P. vivax,* however the other three were negative; of the mixed infection, two were found negative and three were found to have only *P. vivax.* Thirteen out of the 14 samples that were identified as *P. vivax* positive by microscopy but both PCR-NALFIA and RDT negative were positive in the nested PCR. The other sample was negative. In the group of negative samples that were also analysed with the nested PCR, 15 samples (21%) were identified as *P. vivax* positive and no other species were found.

**Table 5:** Overview of results of the field evaluation in Thailand

**Microscopy**	** RDT**	**PCR-NALFIA**				**Total**
		***Negative***	***P. falciparum***	***P. vivax***	***Mixed infection***	
*Negative*		260	3	6	5	274
	*Negative*	257 *	*2*	*6*	*5*	*266*
	*P. falciparum*	*3*	*1*			*4*
*P. falciparum*			32			32
	*P. falciparum*		*32*			*32*
*P. vivax*		14		57	1	72
	*Negative*	*14*		*4*	*1*	*19*
	*P. falciparum*			*1*		*1*
	*P. vivax*			*52*		*52*
*P.f + P.v*			1		1	2
	*P. falciparum*		*1*		*1*	*2*
*P.m + P.v*					1	1
	*Negative*				*1*	*1*
**Total**		274	36	63	8	381

*including four RDT test failures.

**Table 6:** Sensitivity, specificity and agreement of the different diagnostic tests in Thailand

**Detection line**	**Sensitivity (95% CI)**	**Specificity (95% CI)**	**Positive Predictive Value (95% CI)**	**Negative Predictive Value (95% CI)**	**Kappa**
***Overall pan-Plasmodium***					
SD-Bioline *vs* Microscopy (n = 377)	0.813 (0.723-0.879)	0.985 (0.960-0.995)	0.956 (0.884-0.986)	0.930 (0.892-0.956)	0.83
Microscopy *vs* PCR-NALFIA (n = 381)	0.869 (0.787-0.924)	0.949 (0.914-0.970)	0.869 (0.787-0.924)	0.949 (0.914-0.971)	0.82
SD-Bioline *vs* PCR-NALFIA (n = 377)	0.822 (0.734-0.887)	0.989(0.965-0.997)	0.967 (0.900-0.991)	0.934(0.897-0.958)	0.85
***P. falciparum***					
SD-Bioline *vs* Microscopy (n = 377)	1 (0.874-1)	0.985 (0.964-0.994)	0.872 (0.718-0.952)	1 (0.986-1)	0.92
Microscopy *vs* PCR-NALFIA (n = 381)	0.773 (0.617- 0.880)	1 (0.986-1)	1 (0.874-1)	0.971 (0.946-985)	0.88
SD-Bioline *vs* PCR-NALFIA (n = 377)	0.796 (0.642-0.897)	0.988 (0.967-0.996)	0.898 (0.748-0.967)	0.973 (0.948-0.987)	0.84
P. vivax*					
SD-Bioline *vs* Microscopy (n = 338)	0.722 (0.602-0.818)	1 (0.982-1)	1 (0.914-1)	0.930 (0.892-0.956)	0.78
Microscopy *vs* PCR-NALFIA (n = 381)	0.845 (0.735-0.916)	0.951 (0.920-0.972)	0.80 (0.689-0.880)	0.964 (0.935-0.981)	0.78
SD-Bioline *vs* PCR-NALFIA (n = 338)	0.754 (0.633-0.846)	1 (0.982-1)	1 (0.914-1)	0.941 (0.905-0.964)	0.82

*Because the pLDH line on the SD-bioline RDT also detects *P. falciparum,* all *P. falciparum* samples were excluded from the analysis with SD-Bioline.

**Table 7:** Sensitivities of SD-Bioline and PCR-NALFIA compared to microscopy differentiated in parasitaemia

**Parasitaemia**	**SD Bioline**	**PCR-NALFIA**
> 50.000 / μl	100% (3 / 3)	100% (3 / 3)
5000 to 50.000 / μl	100% (49 / 49)	100% (49 / 49)
500 to 5000 / μl	100% (22 / 22)	100% (22 / 22)
100 to 500 / μl	**81.8%** (9 / 11)	100% (11 / 11)
50 to 100 / μl	**66.7%** (2 / 3)	100% (3 / 3)
< 50 / μl	**10.5%** (2 / 19)	**26.3%** (5 / 19)

## Conclusion

This paper describes the development of a multiplex PCR assay for the detection and differentiation of malaria parasites combined with NALFIA to enable fast and easy readout of the results. This assay has been shown to be sensitive and specific and the readout with NALFIA is straightforward.

The assay was evaluated in three different phases. First, the analytical sensitivity and specificity was assessed and found to be very good, although the detection of *P. vivax* was slightly less sensitive than the detection of *P. falciparum* which is likely caused by the lower parasite densities present in a *P. vivax* sample.

Next, the robustness, reproducibility and repeatability of the assay was tested in the ring trial. This phase is often not performed in diagnostic test development and evaluation but it can give extremely valuable results. The current trial has shown, for example, that although in general the reproducibility of the assay was acceptable, there were differences in the results. Laboratory 3 failed to correctly identify some of the samples. Although the overall agreement between the laboratories was good, it was striking that laboratory 3 identified more samples as negative or a mono-infection, pointing to a reduced sensitivity of the assay in this laboratory. The exact mechanism behind this reduced sensitivity in this laboratory was not further investigated and can be the result of a variety of factors altough all reagents, samples and materials were provided to the laboratory and were the same as the other laboratories received. It should be noted that this was the only laboratory that performed the amplification on a Biorad-CFX Real-Time PCR machine, whereas the other laboratories used conventional PCR machines. The Real-Time machinery has a variable ramping time which can be installed manually whereas the other conventional PCRs have fixed ramping times and are therefore comparable in this respect. It was however not investigated whether or not the Real-Time PCR machine was the cause of the slightly poorer results but it cannot be excluded. This issue would not have would not have surfaced if a ring trial had not been performed. In all three laboratories and in the field evaluation, use of the NALFIA as a readout system was received enthusiastically by performers and was carried out without any particular difficulties.

In the final field evaluation, the sensitivity, specificity and predictive value of the assay was studied in a moderate transmission area for *P. falciparum* and *P. vivax* parasites. In this study the results of PCR-NALFIA were compared to both microscopy and RDT. Although microscopy is often considered to be the gold standard, it is not sensitive enough compared to PCR or other molecular diagnostics and may consequently obscure the results of the specificity of the assay under investigation [15]. RDTs are even less sensitive but were used in this study to compare the performance of the PCR-NALFIA with another assay that is used on a day-to-day basis [6]. Because of these limitations the discordant results were also analysed by a sensitive nested PCR. This study showed that PCR-NALFIA performs very well for the detection of *P. falciparum* and is as sensitive and specific as microscopy and PCR. The assay was also very sensitive compared to RDT and was able to pick up all samples over 50 parasites/ul that were found positive in microscopy. For *P. vivax,* the microscopy performed in this study was more sensitive although some samples that were found negative with microscopy were found positive in PCR and confirmed positive with the nested PCR as well. The microscopy performed extremely well in this study and it is therefore essential to mention that the protocol used for slide reading is different from the one that is used as standard in routine care, and which is usually less sensitive.

While it was not observed in the present study, a possible concern might be the risk of contamination when a PCR sample is transferred to the NALFIA device. Altough this could partly be circumvented by performing the PCR set up and amplification in one room and do the read-out in another, a closed system in which no leakage to the environment is possible should be the next step in the development. Another current disadvantage is that this assay relies on PCR technology for amplification, which demands electricity. This does not have to be a problem if the assay is going to be performed in a setting where constant supply of electricity can be guaranteed. However, if one desires to use this assay in more remote settings in which no constant supply of electricity is available, alternative platforms that are less dependent on electricity, such as LAMP [21] or NASBA [22], could be considered. Nevertheless, although these assays are generally considered to be sensitive and specific they should be subjected to a thorough analysis on their robustness, reproducibility, repeatability and user-friendliness before implementation can be considered.

The current NALFIA strips were produced by a manufacturer specialized in the production of lateral flow devices and because of the large amount of strips produced the price for the NALFIA strips is around €0,50 cent. The final costs will depend on the quanitity of devices produced. This will also depend on the shelflife of the NALFIA devices. This has not been extensively studied in this study but the devices used were performing well up to a period of 6 months after dispatching at room temperature.

Even though all reagents and materials were provided by the developers of the assay and the technicians that performed the assay, in both the ring trial and laboratory evaluation, had previous experience in molecular techniques, no training on the current protocol was given. This shows PCR-NALFIA is a user friendly procedure that can be performed with no or little extra training.

The assay in its current format, or slightly modified with more sensitive detection of *P. vivax*, could be an excellent tool for epidemiological studies on prevalence or distribution of parasites. In addition, it could be used as a screening tool at regional level for malaria control programmes especially in countries with declining transmission [23]. Molecular tools have been shown to be especially valuable in areas where there is moderate to little transmission [24] and PCR-NALFIA may be a straightforward method to implement.

## Competing interests

The authors declare that they have no competing interests.

## Authors’contributions

PM coordinatied and and preparated the laboratory and trial work, drafted the protocols, analysed the data and drafted the manuscript. AM participated in the initial test development. LdB prepared the laboratory evaluation and was involved in the single laboratory trial. JF provided technical assistance and prepered the lateral flow devices. JS and LK participated in the field trial and performed expert microscopy of the samples. JV, CvO and RH participated in the ring trial, and critically read the manuscript. BW: perfromed the PCR-NALFIA in the field. SP: provided local coordination and supervision of the fieldwork in Thailand, participated in data collection, entry and analysis of the data and critical reading of the manuscript. HS: Conceived the study and coordinated the Malactres project, drafted the of protocols, and was involved in design, preperation and supervision of laboratory and field trials, Coordinaton of the MALACTRES project. AA: Supervised the initial test development. All authors read and approved the final manuscript.
